# Salient sounds distort time perception and production

**DOI:** 10.3758/s13423-023-02305-2

**Published:** 2023-07-10

**Authors:** Ashley Symons, Fred Dick, Adam Tierney

**Affiliations:** 1 Department of Psychological Sciences, Birkbeck, University of London, Malet Street, London, WC1E 7HX UK; 2 Experimental Psychology, Division of Psychology and Language Sciences, University College London, London, UK

**Keywords:** Auditory salience, Attention, Synchronization, Timing

## Abstract

The auditory world is often cacophonous, with some sounds capturing attention and distracting us from our goals. Despite the universality of this experience, many questions remain about how and why sound captures attention, how rapidly behavior is disrupted, and how long this interference lasts. Here, we use a novel measure of behavioral disruption to test predictions made by models of auditory salience. Models predict that goal-directed behavior is disrupted immediately after points in time that feature a high degree of spectrotemporal change. We find that behavioral disruption is precisely time-locked to the onset of distracting sound events: Participants who tap to a metronome temporarily increase their tapping speed 750 ms after the onset of distractors. Moreover, this response is greater for more salient sounds (larger amplitude) and sound changes (greater pitch shift). We find that the time course of behavioral disruption is highly similar after acoustically disparate sound events: Both sound onsets and pitch shifts of continuous background sounds speed responses at 750 ms, with these effects dying out by 1,750 ms. These temporal distortions can be observed using only data from the first trial across participants. A potential mechanism underlying these results is that arousal increases after distracting sound events, leading to an expansion of time perception, and causing participants to misjudge when their next movement should begin.

**Supplementary Information:**

The online version contains supplementary material available at 10.3758/s13423-023-02305-2.

## Introduction

Acoustic environments are complex, presenting a steady stream of interruptions that can interfere with goal-directed behavior. In a coffee shop, for example, you must focus on clearly communicating your order while ignoring a dozen conversations, traffic noise, the hiss of the espresso machine, and miscellaneous electronic hums. Although distraction is a common experience, we know surprisingly little about how sound affects ongoing behavior: what factors cause a sound to disrupt behavior, how rapid is this disruption, and how long does the interference last?

In the visual system, researchers can track eye movements to measure attentional capture by parts of a visual scene (Foulsham & Underwood, 2008). Using eye-tracking data as ground truth, researchers have built computational models of visual salience in which several feature maps with center-surround inhibition are combined to form a “salience map” (Itti et al., 1998). Researchers studying auditory salience have created similar maps in time–frequency “space” (Duangudom & Anderson, 2007; Kalini & Narayanan, 2007; Kaya & Elhilali, 2012; Kayser et al., 2005). An alternate approach is to track feature-specific deviance from prediction relative to local and longer-term statistics (Kaya & Elhilali, 2014; Tsuchida & Cottrell, 2012).

These auditory salience models have been validated by relating model predictions to subjective ratings (Duangudom & Anderson, 2007; Huang & Elhilali, 2017; Kalini & Narayanan, 2007; Kaya & Elhilali, 2014; Kayser et al., 2005; Kim et al., 2014; Tsuchida & Cottrell, 2012; Zhao et al., 2019). Research has also investigated effects of sound presentation on performance in a difficult unrelated task, such as short-term memory retrieval (Jones et al., 2000; Little et al., 2010; Röer et al., 2015; Schlittmeier et al., 2012) or perceptual detection (Southwell et al., 2017). This research has demonstrated that auditory scenes and objects featuring a greater degree of spectrotemporal modulation are rated as more salient and interfere with task performance to a greater degree. However, because these techniques cannot precisely measure the time course of behavioral disruption, it has not been possible to test several key predictions made by auditory salience models. First, they predict that ongoing goal-directed behavior will be disrupted following points in time that feature a high degree of spectrotemporal modulation (although they do not typically make specific predictions regarding the time course of disruption). To test this prediction, behavioral disruption measurements must be precisely time-locked to sound events. Second, they predict that different auditory features are combined into an overarching saliency map, and that therefore changes in different dimensions (such as amplitude and pitch) disrupt behavior via the same mechanism. This prediction can be tested in part by examining the time course of behavioral disruption: If different auditory features capture attention via overlapping mechanisms, then the latency and duration of the disruption should be similar across dimensions.

Some prior evidence supporting these predictions comes from research on physiological components of the orienting response (Sokolov, 1963). The onset of loud sounds, for example, leads to time-locked changes in pupil dilation and the galvanic skin response, compared with the onset of soft sounds (Antikainen & Niemi, 1983; Barry, 1975; Liao et al., 2016; but see Stelmack & Siddle, 1982). Sudden shifts in frequency have also been linked to changes in pupil size, with greater dilation for larger shifts (Bala & Takahashi, 2000; Marois et al., 2018; Wetzel et al., 2016). Stimuli with greater high-frequency amplitude modulation (roughness) have been linked to microsaccadic inhibition (Zhao et al., 2019). Similarly, changes in intensity (Rinne et al., 2006) and frequency (e.g., Berti et al., 2004; Escera et al., 1998; Getzmann et al., 2022; Schröger & Wolff, 1998) elicit an increase in event-related potential (ERP) amplitudes approximately 300 ms (P3a response) after the onset of the change in auditory oddball paradigms. This response increases as a function of the magnitude of stimulus change (Berti et al., 2004; Rinne et al., 2006). Novel or unexpected sounds outside the focus of attention also elicit a P3a response (e.g., Bidet-Caulet et al., 2015; Bigliassi et al., 2018; Wetzel et al., 2013), which correlates with subjective ratings of arousal (early P3a; Masson & Bidet-Caulet, 2019). Accordingly, models of involuntary attention suggest that the P3a provides an index of involuntary orienting of attention (Escera et al., 1998; Schröger & Wolff, 1998; Wetzel & Schröger, 2014). Task-irrelevant sound changes have also been linked to decreased phase-locking and gamma responses to task-relevant sounds (Huang & Elhilali, 2020). Unlike the behavioral measures of attentional capture reviewed above, the orienting response is precisely time-locked to stimulus features; however, it not yet has been clearly linked to an immediate behavioral disruption. Based on these physiological studies, we hypothesize that sudden acoustic changes are rapidly followed by a disruption of ongoing behavior, but that individuals can rapidly recover from this interference.

**Fig. 1:**
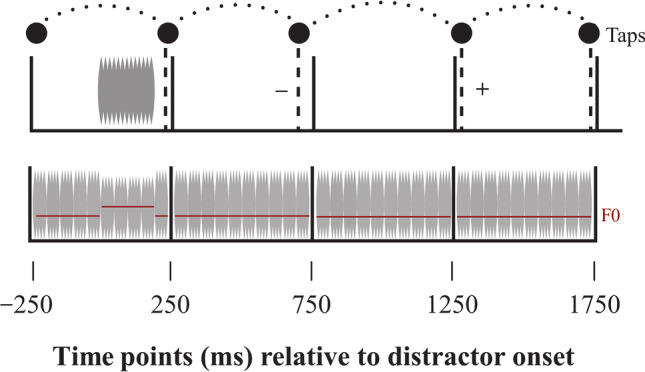
Schematic depiction of click sequence and distractor sounds in Experiments 1 & 2 (top) and Experiment 3 (bottom). Click sequences were presented at a rate of 2 Hz with auditory distractors presented at random intervals between clicks. Distractor sounds always occurred 250 ms after the previous click and lasted for a duration of 200 ms. Participants tapped to the beat of the clicks while ignoring the distractor sounds. Tapping asynchrony was computed as the difference between participants’ tap time and the nearest click time for the four clicks following the distractor (250 ms, 750 ms, 1,250 ms, 1,750 ms). Tapping earlier than the beat corresponded to a negative value. Tapping later than the beat corresponded with a positive value. Change in asynchrony was computed as the difference in tap-click asynchrony at each time point compared with the previous time point

We conducted six experiments on short-term attentional capture. Due to the COVID-19 pandemic, three experiments were initially conducted online, with three subsequent matched in-lab replications. We tested the prediction that acoustic edges, or abrupt boundaries in waveform amplitude and spectral envelope, would lead to rapid, transient disruption in ongoing behavior. Participants tapped to a click track while task-irrelevant sounds or pitch changes occurred occasionally between clicks (Fig. 1). This task samples behavioral disruption at the tapping rate (2 Hz), enabling investigation of its time course. Specifically, attentional capture was sampled at 250, 750, 1,250, and 1,750 ms after distractor presentation. Presenting distractors exactly halfway between clicks ensured that participants would not perceptually integrate the distractor and click onsets, given that prior research has shown that there is a fixed temporal window for onset integration less than 120 ms in duration (Repp, 2004). We predicted that distractor presentation would disrupt performance within the first 2 s following the onset of the distractor, pulling participants’ taps away from the click track. Further, we predicted that this disruption would be greater for more salient distractors. Specifically, we predicted that tapping disruption would be greater for rough versus smooth distractors (Experiments 1A and 1B), loud versus soft distractors (Experiments 2A and 2B), and large versus small pitch changes (Experiments 3A and 3B).

## Materials and methods

### Participants

All online participants were recruited through Prolific. The experiments were conducted via the online experiment platform Gorilla, and all participants were asked to wear headphones. Automated procedures ensured that participants were using the Google Chrome web browser on a computer. The Ethics Committee in the Department of Psychological Sciences at Birkbeck, University of London, approved all experimental procedures. Informed consent was obtained from all participants. Participants were compensated for their participation in the form of payment at a standard rate. For each participant, trials in which the maximum tapping asynchrony was more than ± 250 ms were excluded. For online studies, participants with fewer than 36 remaining trials were excluded from analysis.

A total of 101 online participants (39 female, 61 male, 1 prefer not to report) between the ages of 18 and 40 (mean age = 24.19 years, *SD* = 5.79) took part in Experiment 1A, with a final sample of 67 participants (29 female, 37 male, 1 prefer not to report, mean age = 24.61, *SD* = 6.01). Twenty-one in-person participants (10 female, 11 male) between the ages of 22 and 52 (mean age = 33 years, *SD* = 7.13) took part in Experiment 1B. Sixty-two online participants (19 female, 43 male) between the ages of 18 and 52 (mean age = 26.03 years, *SD* = 6.82) took part in Experiment 2A, with a final sample of 49 participants (16 female, 33 male, mean age = 26.69 years, *SD* = 7.21). Twenty in-person participants (3 female, 16 male, one other gender) between the ages of 20 and 42 (mean age = 31.65 years, *SD* = 7.67) took part in Experiment 2B. Fifty-eight online participants (28 female, 29 male, one other gender) between the ages of 18 and 52 (mean age = 26.90 years, *SD* = 6.13) took part in Experiment 3A, with a final sample of 52 participants (26 female, 25 male, 1 other gender mean age = 26.92 years, *SD* = 6.28). The same participants who took part in Experiment 1B also took part in Experiment 3B.

### Stimuli

In Experiments 2A and 3A, stimuli consisted of forty-two 2-Hz isochronous click sequences that were each 8 s in duration, with distractor sounds presented in one of seven positions (after the 6th through 12th clicks for Experiment 2A and after the 5th through 11th clicks for Experiment 3A). In Experiments 1A, 1B, 2B, and 3B, stimuli consisted of two 157.5-s 2-Hz isochronous click sequences, each of which contained 40 distractor sounds pseudorandomly located such that there were at least six clicks between successive distractor sounds. No clicks occurred during the first 10 clicks (5 s) of the stimulus or during the last 6 clicks (3 s) of the sequence. For Experiments 1B, 2B, and 3B stimuli were presented using PsychToolbox (Version 3.0.17) run in MATLAB (MathWorks, Inc), and the sound was delivered via Sennheiser HD 25–1 ii headphones, with the maximum dB level kept at 75 dB SPL.

In each experiment, clicks were broadband impulses spanning 10 time points (0.23 ms in duration, 44,100-Hz sample rate) with a condensation polarity. Clicks had an equal amplitude to the distractors in Experiments 1 and 2 but had a relative peak amplitude of + 0.3 relative to the distractors in Experiment 3 to ensure that the clicks were clearly audible against the continuous background sounds. Distractors were 200 ms in duration with an onset beginning 250 after the previous click. In Experiments 1A and 1B, distractors were white noise carriers with a 10-ms cosine on/off ramp and amplitude modulated deeply (100%) or shallowly (20%) at 60 Hz. In Experiments 2A and 2B, distractors were a pneumatic drill sound (Zhao et al., 2019), which was presented at a greater versus lesser amplitude (RMS dB difference = 19.17). In Experiments 3A and 3B, a tone sequence was presented at a rate of 20 Hz alongside the click track. The 50-ms pure tones (10-ms on/off ramp) in the sequence had a fundamental frequency of 440 Hz. Within each tone sequence there was a pitch deviation of either + 1 semitone or + 6 semitones that lasted for 200 ms (i.e., four 50-ms tones). Importantly, although one semitone is a small change relative to the large change condition, it is well above most individuals’ detection threshold (Kidd et al., 2007). The amplitude of the deviant pitch tones was reduced to balance the relative loudness of the tones using the stationaryLoudness function in MATLAB (Stephen Hales Swift, 2020).

### Procedure

For Experiments 1A, 2A, and 3A, participants were provided with on-screen task instructions asking them to tap along with the clicks by pressing the ‘j’ key on the keyboard while ignoring a distracting sound. Participants were provided with an example of the click sequence without the distractor so that they could practice tapping along to the beat. For Experiments 1B, 2B, and 3B, participants were provided with verbal task instructions asking them to tap on a microphone along with the clicks. Experiments 2A and 3A lasted approximately 20 min, while Experiments 1A, 1B, 2B, and 3B lasted approximately 7–8 min.

### Data processing and analysis

For Experiments 1A, 2A, and 3A, sound timing information and participant response times were automatically recorded. For Experiments 1B, 2B, and 3B, a stereo file was recorded with the stimulus in one channel and the participants’ response in the other channel. Custom MATLAB scripts detected click and tap onsets by setting a threshold and relaxation time. Onsets were marked when the amplitude exceeded threshold and the amount of time elapsed since the last onset exceeded the relaxation time. Thresholds and relaxation times were adjusted on a participant-by-participant basis to ensure that each click and tap was marked and that no onsets were erroneously detected.

Since the click onset was the behavioral target (i.e., the event to which participants were attempting to align their movements), the difference between the participant’s tap time and the time of nearest click onset was recorded. For online Experiments 1A, 2A, and 3A, the true asynchrony between tap times and click onsets could not be measured reliably due to variations in sound onset latency resulting from differences in computer setups across participants. To create a measure of timing which was reliable and valid across both online and in-lab experiments, we therefore measured the change in tap-click asynchrony by calculating the difference between the asynchrony at each click onset and the asynchrony at the previous click onset. For example, the change in asynchrony at 750 ms was computed as the tap-click asynchrony at the click occurring 750 ms after the distractor minus the tap-click asynchrony at the click occurring 250 ms after the distractor. This procedure normalizes any cross-participant or cross-trial differences in latency. Change in asynchrony was assessed for the four clicks following distractor presentation, which occurred at the following time points relative to distractor onset: + 250 ms; + 750 ms; + 1,250 ms; and + 1,750 ms.

Since the data were not normally distributed and observations at each time point not independent, nonparametric statistics were used. Our analysis approach was motivated by specific hypotheses based on the initial pattern of responses. First, we hypothesized that there would be a significant change in asynchrony following the distractor. To test this hypothesis, Wilcoxon signed rank tests were conducted, comparing the change in asynchrony to zero at each of the four offset intervals after collapsing across conditions. We then hypothesized that this effect would be modulated by the magnitude of stimulus change. To test this hypothesis, we conducted Wilcoxon signed rank tests comparing conditions of high versus low magnitude of stimulus change, again at each offset interval. *P*-values were FDR-corrected given that comparisons were run across the four time points. To examine performance on the first trial, data from the first trial was extracted and Wilcoxon signed rank tests used to determine whether there was a change in overall asynchrony relative to zero at each time point. One participant in Experiment 3B who tapped at antiphase following the first distractor was excluded from this analysis. For all experiments, only time points with significant effects are reported (for all other time points, p_corrected_ > 0.05). Analysis of tapping variability was also conducted but was not modulated by salience, and therefore is not included in the results. However, these data and analyses are available in the online repository. Statistics for all comparisons along with the number of trials included in each experiment can be found in the Supplementary Materials.

## Results

### Roughness

**Fig. 2:**
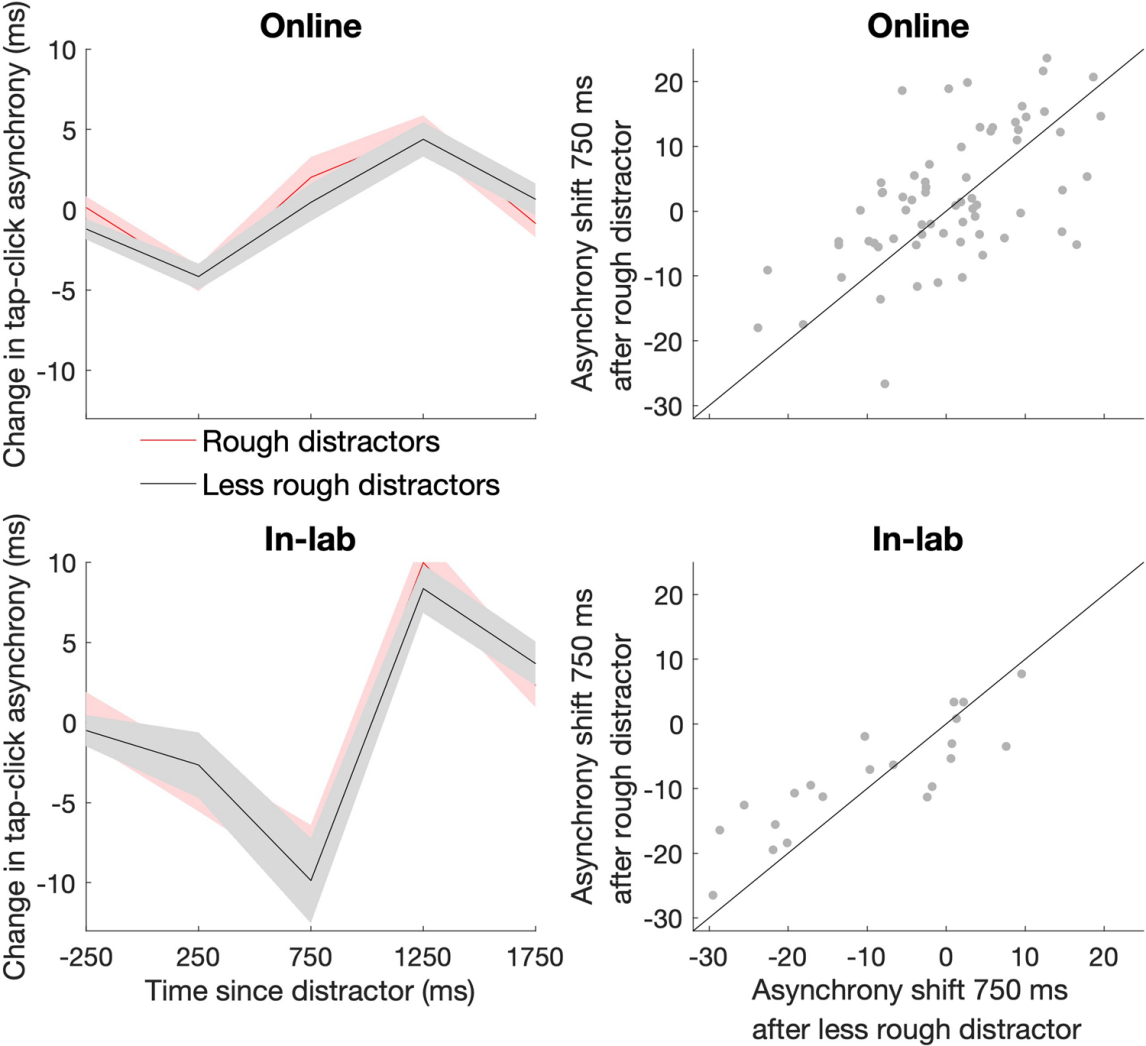
Effects of task-irrelevant sounds on auditory-motor timing are not modulated by distractor roughness. (Left) Mean change in tap-click asynchrony after distractor presentation in online study (top) and in-lab replication (bottom). Distractors were either rough (100% modulation at 60 Hz, red lines) or less rough (20% modulation at 60 Hz, black lines). The shaded region indicates the standard error of the mean. (Right) Scatterplot displaying relationship between the asynchrony shift after rough and less rough distractors in online study (top) and in-lab replication (bottom). The line displays the identity function y = *x*. (Colour figure online)

In Experiments 1A (online) and 1B (in-lab), we measured the effect of distractor roughness on attentional capture (Fig. 2). Collapsing across the more rough and less rough conditions, we found an initial shift towards earlier tapping (online, at 250 ms, *z* =  − 5.10, p_corrected_ < 0.001; in-lab, at 250 ms, *z* =  − 2.49, p_corrected_ = 0.013 and at 750 ms, *z* =  − 3.11, p_corrected_ = 0.004), followed by a slowing down of tapping at 1,250 ms (online, *z* = 4.53, p_corrected_ < 0.001; in-lab, *z* = 3.98, p_corrected_ < 0.001). For the in-lab experiment, this slowing down persisted to 1,750 ms (*z* = 2.69, p_corrected_ = 0.009). There was, however, no difference between the more and less rough distractors at any time point (p_corrected_ > 0.05).

**Fig. 3:**
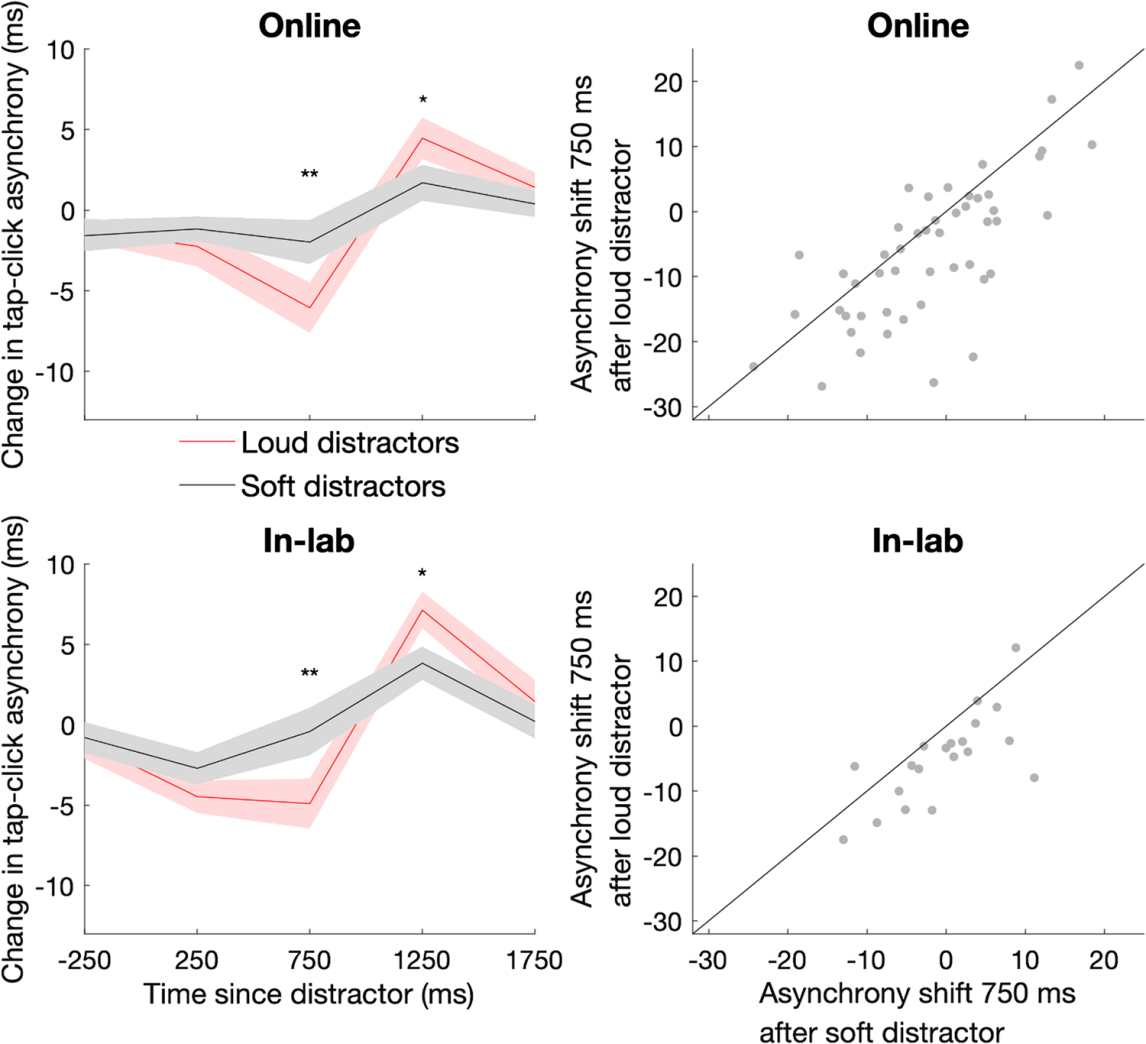
Effects of task-irrelevant sounds on auditory-motor timing are modulated by distractor volume. (Left) Mean change in tap-click asynchrony after distractor presentation in online study (top) and in-lab replication (bottom). Distractors were either loud (red lines) or soft (black lines; rms dB difference = 19.17). ** indicates time points at which p_corrected_ < 0.01, * indicates time points at which p_corrected_ < 0.05. The shaded region indicates the standard error of the mean. (Right) Scatterplot displaying relationship between the asynchrony shift after soft and loud distractors in online study (top) and in-lab replication (bottom). The line displays the identity function *y* = *x*. (Colour figure online)

### Volume

In Experiments 2A (online) and 2B (in-lab), we measured the effect of distractor volume on attentional capture (Fig. 3). Collapsing across the loud and soft conditions, we found an initial shift towards earlier tapping (online, at 250 ms, *z* =  − 2.13, p_corrected_ = 0.044 and at 750 ms, *z* =  − 2.78, p_corrected_ = 0.011; in-lab, at 250 ms, *z* =  − 3.66, p_corrected_ = 0.001), followed by a slowing down of tapping at 1,250 ms (online, *z* = 3.06, p_corrected_ = 0.009; in-lab, *z* = 3.92, p_corrected_ < 0.001). In both experiments the loud distractor led to earlier tapping at 750 ms compared with the soft distractor (online, median difference = 4.6 ms, *z* =  − 3.24, p_corrected_ = 0.005; in-lab, median difference = 4.6 ms, *z* =  − 3.25, p_corrected_ = 0.005). Across both experiments, the loud distractor led to later tapping at 1,250 ms relative to the soft distractor (online; *z* = 2.46, p_corrected_ = 0.028; in-lab, *z* = 2.58, p_corrected_ = 0.020).

### Pitch change

**Fig. 4:**
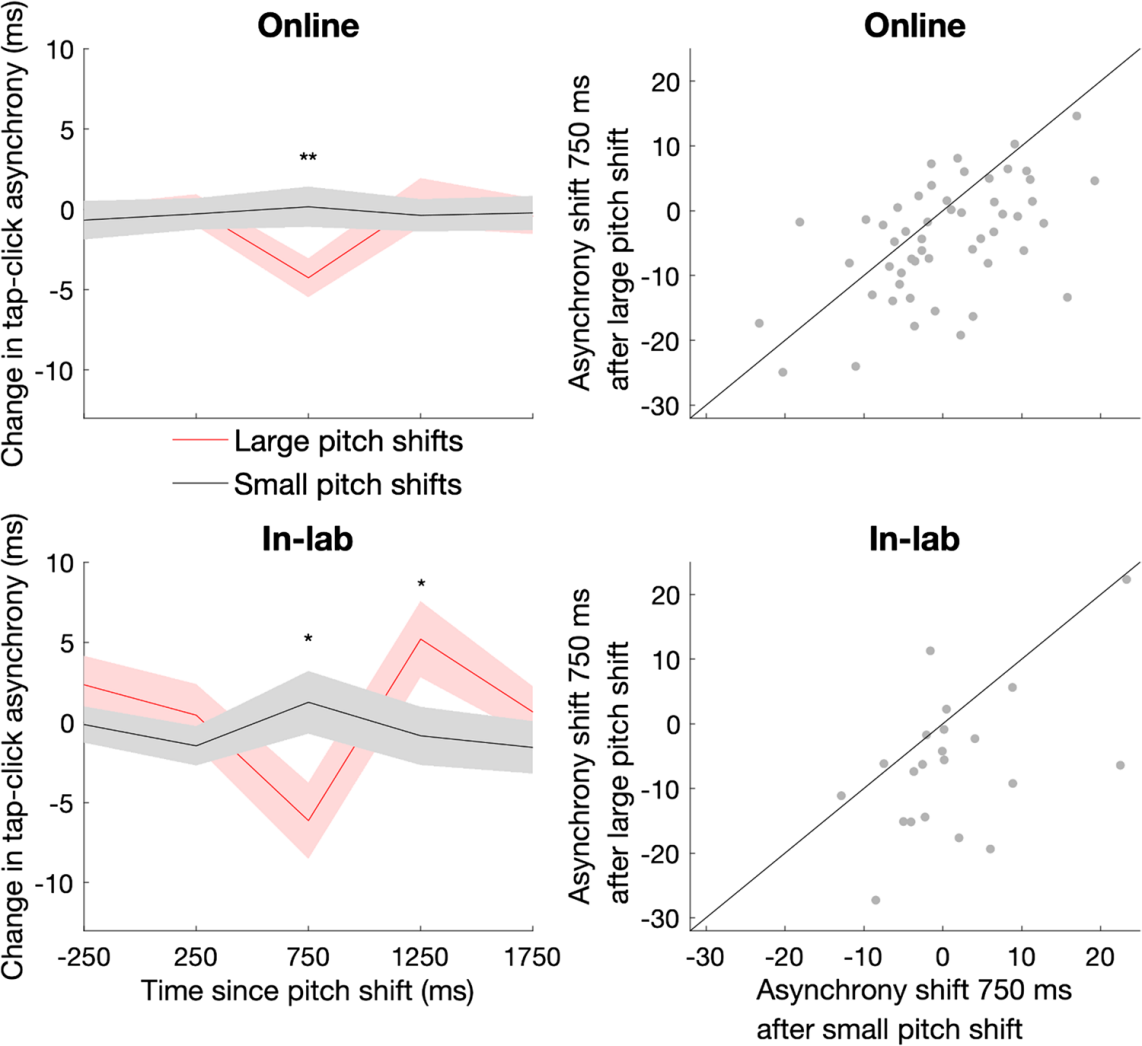
Effects of task-irrelevant pitch changes on auditory-motor timing are modulated by change magnitude. (Left) Mean change in tap-click asynchrony after change in pitch of a constant background sound in online study (top) and in-lab replication (bottom). Pitch changes were either large (6 st, red lines) or small (1 st, black lines). ** indicates time points at which p_corrected_ < 0.01, * indicates time points at which p_corrected_ < 0.05. The shaded region indicates the standard error of the mean. (Right) Scatterplot displaying relationship between the asynchrony shift after large and small pitch changes in online study (top) and in-lab replication (bottom). The line displays the identity function *y* = *x*. (Colour figure online)

In Experiments 3A (online) and 3B (in-lab) we measured the effect of the magnitude of pitch change on attentional capture (Fig. 4). Collapsing across the large and small pitch change conditions, we found no overall effect of distractor presentation on tapping asynchrony (all p_corrected_ > 0.05). However, the large pitch shift led to earlier tapping at 750 ms compared with the small pitch shift (online, median difference 2.1 ms, *z* =  − 3.29, p_corrected_ = 0.004; in-lab, median difference 6.2 ms, *z* =  − 2.90, p_corrected_ = 0.015). In the in-lab experiment, the large pitch shift led to later tapping at 1250 ms (*z* = 2.28, p_corrected_ = 0.046).

### Individual differences

Overall, these findings were broadly replicable across the online and in-lab experiments: distractor presentation was linked to an initial shift towards earlier tapping followed by a decrease in tempo, with the magnitude of the initial shift modulated by volume and pitch shift magnitude but not roughness. However, although clear tapping shifts were visible for most individual participants (Fig. S1), there was considerable variability across participants in the timing and extent of tapping shifts. To determine whether there existed reliable individual differences in attentional capture, for each experiment we used Spearman’s correlations to compare the size of the tapping shift at each time point across the two conditions. Across all six experiments, the resulting correlations were strongest at 750 ms, and so we focus on this time point here. Cross-condition correlations at 750 ms were, for Experiment 1A, rho = 0.56, *p* < 0.001; for Experiment 1B, rho = 0.88, *p* < 0.001; for Experiment 2A, rho = 0.70, *p* < 0.001; for Experiment 2B, rho = 0.73, *p* < 0.001; for Experiment 3A, rho = 0.51, *p* < 0.001; and for Experiment 3B, rho = 0.39, *p* = 0.081.

### Single-trial data

**Fig. 5:**
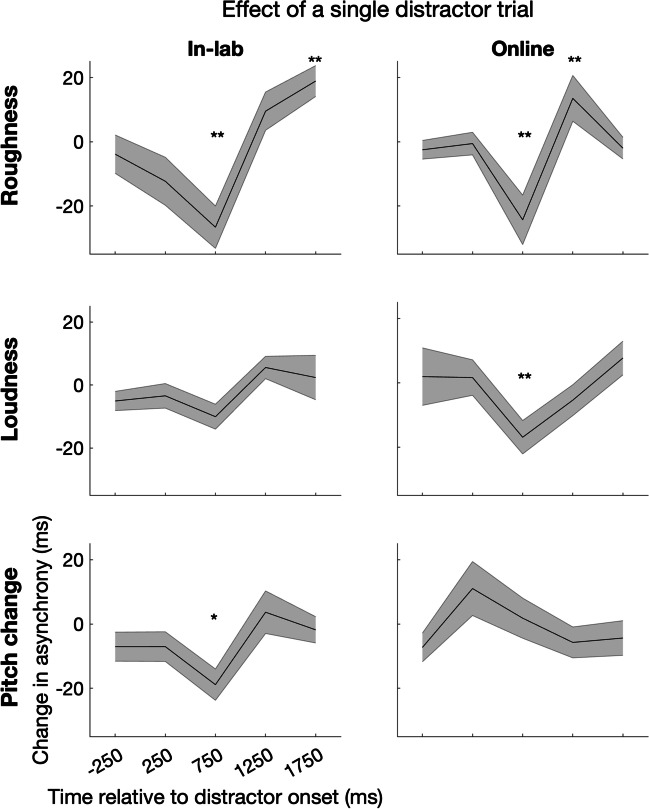
Effects of the first distractor on auditory-motor timing across roughness (top), loudness (middle), and pitch change (bottom) experiments. Each plot shows the mean change in tap-click asynchrony after distractor onset in the online experiments (left) and in-lab replication (right). ** indicates time points at which p_corrected_ < 0.01, * indicates time points at which p_corrected_ < 0.05. The shaded region indicates the standard error of the mean

To determine whether this paradigm could be used to examine the dynamics of attentional capture on a trial-by-trial basis (e.g., for events whose salience may diminish following repeated exposure), we tested whether there was a reliable change in asynchrony following the first presentation of the distractor in each experiment, collapsed across conditions. Across multiple experiments, the effect of the distractor on tapping synchronization could be observed within a single trial (Fig. 5). In Experiments 1A and 1B, there was a shift towards earlier tapping on the first trial at 750 ms (Wilcoxon signed rank test; online, *z* =  − 4.66, p_corrected_ < 0.001; in-lab, *z* =  − 3.18, p_corrected_ = 0.003) followed by a slowing down of tapping (online, 1,250 ms, *z* = 3.461, p_corrected_ = 0.001; in-lab, 1,750 ms, *z* = 3.29, p_corrected_ = 0.003). In Experiments 2A and 2B, there was a significant shift towards earlier tapping on the first trial at 750 ms in the online (*z* =  − 2.99, p_corrected_ = 0.011) but only a trend in the in-lab experiment (*z* =  − 1.94, p_corrected_ > 0.05). In Experiments 3A and 3B, there was a shift towards earlier tapping following the first pitch change at 750 ms in the in-lab experiment (*z* =  − 2.52, p_corrected_ = 0.047) but not in the online experiment (z =  − 0.046, p_corrected_ > 0.05).

## Discussion

Our results confirm a key hypothesis made by computational models of auditory salience (Duangudom & Anderson, 2007; Kalini & Narayanan, 2007; Kaya & Elhilali, 2012, 2014; Kayser et al., 2005; Tsuchida & Cottrell, 2012)—namely, that acoustic edges will be salient. Here, we show that acoustic onsets and pitch changes cause rapid, transient disruption in ongoing synchronized tapping behavior, with greater effects for more salient sounds. Models of auditory salience also suggest different features are combined into a single overarching salience map. We find that the task disruption across experiments follows a highly similar time course, with effects of sound volume and pitch shift size appearing at 750 ms and no longer evident by 1,750 ms, potentially suggesting that different types of sound events disrupt behavior via overlapping mechanisms (see further discussion below). The time course of the performance impairment also roughly parallels prior reports of physiological and electroencephalographic effects of salient sound presentation, suggesting a link between the orienting response and disruption of goal-directed behavior. For example, evidence from ERPs suggests that salient distractors elicit an orienting response at approximately 200–350 ms followed by a reorienting response between 400–600 ms relative to distractor onset (Berti et al., 2004; Bidet-Caulet et al., 2015; Escera et al., 1998; Getzmann et al., 2022; Rinne et al., 2006; Schröger & Wolff, 1998); this broadly aligns with the time course of behavioral disruption in the present experiments. However, effects with a similar time course might nonetheless be underpinned by distinct mechanisms. Future work directly linking this tapping shift with physiological measures of arousal is needed to determine the precise mechanisms underpinning this effect. This could be achieved by correlating individual differences in the magnitude of the tapping shift with increases in pupil dilation responses or P3 amplitudes in response to salient sound changes. Future work comparing the magnitude of the tapping shift across within-subject salience manipulations with model-based saliency maps could provide further support for the idea that the similarity in the time course of the tapping shift across acoustic features reflects a common mechanism. Here we would predict that the magnitude of the tapping shift would correlate with saliency as predicted by the model. Moreover, if features such as frequency and intensity are combined into an overarching saliency map, we would predict that responses to a combination of features (e.g., a change in frequency and intensity) would produce a larger tapping shift compared with a change in a single feature.

Not only was synchronization impaired following distractor presentation, but it was systematically biased: Participants’ tapping shifted to be earlier in time. This suggests that they overestimated the passage of time, misjudging when the next click was about to arrive, and therefore planning to move too early. What mechanism could explain this bias? One possibility is that salient sound presentation leads to an increase in arousal, speeding up the rate of internal pacemakers (Gibbon et al., 1984). Prior research has found that time perception expands after experimental manipulations designed to increase arousal, including modulation of body temperature (Wearden & Penton-Voak, 2007), presentation of click trains (Droit-Volet, 2010; Penton-Voak et al., 1996; Wearden et al., 1999), flickering of visual stimuli (Droit-Volet & Wearden, 2002; Ortega & López, 2008), and emotional intensity (Droit-Volet & Meck, 2007). This effect of arousal on the internal pacemaker may also lead to the filled duration illusion, in which filled intervals are perceived as longer than unfilled intervals (e.g., Buffardi, 1971; Ortega & López, 2008; Wearden et al., 2007). Our results can be interpreted in line with the filled duration illusion, in which ‘filled intervals’ (intervals containing distractors) are perceived as longer than unfilled intervals. However, in prior studies, time perception was measured using explicit behavioral judgments which required participants to encode, remember, and compare temporal intervals to standards. Interestingly, the degree of temporal distortion we report here (1% of the interval being timed) is far lower than that reported in previous studies of temporal distortion due to manipulation of arousal. Presentation of click trains, for example, can distort time by as much as 10% (Penton-Voak et al., 1996). This discrepancy in effect magnitude between the current and previous findings suggests that arousal may distort time perception at both encoding and later stages such as retrieval, with distortion at later stages possibly being more severe than at earlier stages.

An alternate explanation for our findings is that synchronization to a metronome requires neural entrainment to the target rhythm (Large & Riess Jones, 1999), and that presentation of a distractor in between clicks briefly interferes with rhythmic temporal expectation (Auksztulewicz et al., 2019). This explanation could account for the transient nature of the distractor effect, as the constant reinforcement of the metronome beat would enable participants to rapidly re-entrain after perturbation (Repp, 2002). This account, however, would have difficulty accounting for the fact that participants’ tapping consistently shifted to be earlier. Since distractors were presented exactly halfway between clicks, an entrainment perturbation account would have no reason to predict that tapping would shift in one direction versus the other. Another possible explanation for this shift to earlier tapping is that the distractor itself triggered a motor response. We consider this explanation unlikely given that the tapping shift typically occurs 750 ms following the distractor, substantially later than simple reaction times auditory stimuli (e.g., Fry, 1975). This explanation could be ruled out by comparing response times to the distractors with the time course tapping shift. We expect that response times would precede and show no correlation with the tapping shift.

Individual differences in the magnitude of timing shifts due to distractor presentation were highly reliable, with cross-condition correlations reaching as high as 0.88 in one experiment. This suggests that there are large, stable differences between participants in the extent of attentional capture due to presentation of task-irrelevant sounds. The source of these individual differences is an interesting target for future research. One possibility, for example, is that participants with greater inhibitory control may be better able to inhibit the capture of attention by task-irrelevant sounds. Individual differences were also observed in the latency of the effect (Fig. S1), potentially accounting for the differences in online and in-lab roughness experiments. However, this difference could have also been explained by online participants reducing the volume setting (amplitude) on their computer due to the slightly aversive nature of the rough sounds.

Auditory roughness is a common feature of natural alarm signals (Arnal et al., 2015), and previous reports have demonstrated a relationship between auditory roughness and both salience ratings and microsaccadic inhibition (Zhao et al., 2019). Nevertheless, we did not find a significant effect of roughness on attentional capture. One possible explanation is that both the high roughness and low roughness stimuli presented in these experiments began with an aversive sharp, wide-band increase in amplitude. This sudden spectrotemporal change may have disrupted behavior robustly enough that the consequences of the subsequent amplitude modulation could not be detected due to ceiling effects. A ceiling effect for rough sounds could also explain why rough distractors elicited the largest tapping shift.

The measure of behavioral disruption presented here has considerable methodological advantages over other similar measures. A single highly reliable measurement can be collected in 3–4 min. The task can be completed online, with similar results from online and in-lab experiments. A significant effect can be captured in a single trial, making possible analysis of changes in salience over time (for example, resulting from stimulus repetition). The task is simple and measures a natural behavior (Savage et al., 2015); as a result, it can be performed by virtually anyone over the age of 4 (Kirschner & Tomasello, 2009). The task does not rely on assessment of performance as correct or incorrect, and therefore is not susceptible to ceiling or floor effects. Finally, the task is a direct measure of behavioral disruption, and unlike salience ratings does not rely on participants’ interpretation of task instructions. This measure, therefore, could be an ideal tool for resolving conflicting predictions made by competing theories of auditory salience. Theoretical models of auditory salience, for example, differ on whether salience is primarily driven by local center-surround contrast (Duangudom & Anderson, 2007; Kalini & Narayanan, 2007; Kaya & Elhilali, 2012; Kayser et al., 2005) versus tracking of statistics on a longer time scale (Kaya & Elhilali, 2014; Tsuchida & Cottrell, 2012). This issue could be investigated by determining whether the temporal distortion response can be suppressed when task-irrelevant sounds are fully predictable.

### Supplementary Information

Below is the link to the electronic supplementary material.

Supplementary file1 (DOCX 696 KB)

## Data Availability

Processed data are available (https://osf.io/x8nhm/). Raw data are available on request from the corresponding author.
