# Supplementary material for: Salient sounds distort time perception and production
Source: Psychon Bull Rev. 2023 Jul 10;31(1):137–47. doi: 10.3758/s13423-023-02305-2 (PMC10866776; doi:10.3758/s13423-023-02305-2)
Supplement: Supplementary file 1 — Supplementary file1 (DOCX 696 KB) [file 13423_2023_2305_MOESM1_ESM.docx]

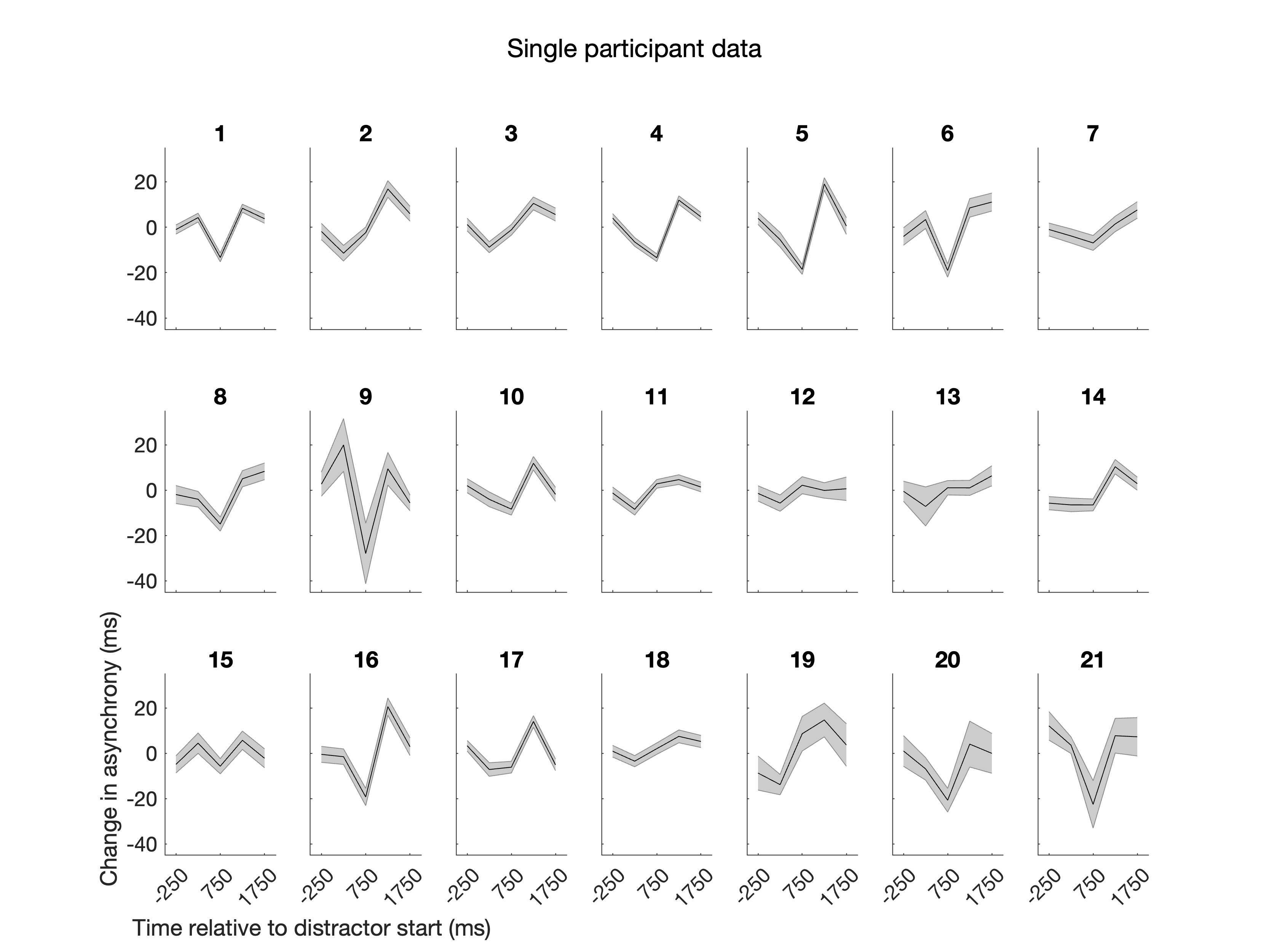


**Figure S1.** Effect of task-irrelevant sounds on audio-motor timing in single participants. Mean changes in tap-click asynchrony after distractor presentation are displayed for each of the 21 participants in Experiment 2. The shaded region indicates the standard error of the mean across trials.

|  |  | # Subjects | Min | Max | Median | Total |
| --- | --- | --- | --- | --- | --- | --- |
| Exp 1a | Rough distractor | 67 | 36 | 40 | 39 | 2610 |
|  | Less rough distractor | 67 | 36 | 40 | 40 | 2620 |
| Exp 1b | Rough distractor | 21 | 33 | 40 | 40 | 828 |
|  | Less rough distractor | 21 | 34 | 40 | 40 | 826 |
| Exp 2a | Loud distractor | 49 | 37 | 42 | 42 | 2002 |
|  | Soft distractor | 49 | 37 | 42 | 42 | 2016 |
| Exp 2b | Loud distractor | 20 | 38 | 40 | 40 | 798 |
|  | Soft distractor | 20 | 39 | 40 | 40 | 797 |
| Exp 3a | Large pitch shift | 52 | 37 | 42 | 42 | 2122 |
|  | Small pitch shift | 52 | 36 | 42 | 41 | 2119 |
| Exp 3b | Large pitch shift | 21 | 31 | 40 | 40 | 821 |
|  | Small pitch shift | 21 | 34 | 40 | 40 | 824 |

**Table 1.** Number of trials included in each experiment and condition including the minimum, maximum, median, and total number of trials (sum across all participants).

**Table 2**. One-sample Wilcoxon tests comparing the change in tapping asynchrony in each condition and time point to zero for Experiments 1A and 1B.

|  | Time Point | B | W | z | p | p (corrected) |
| --- | --- | --- | --- | --- | --- | --- |
| Experiment 1A (online) | 250 | 67 | 321.5 | -5.107 | 0.000 | 0.000 |
|  | 750 | 67 | 1304.5 | 1.034 | 0.301 | 0.402 |
|  | 1250 | 67 | 1863.5 | 4.526 | 0.000 | 0.000 |
|  | 1750 | 67 | 1084.0 | -0.344 | 0.731 | 0.731 |
| Experiment 1B (in-lab) | 250 | 21 | 44 | -2.485 | 0.013 | 0.013 |
|  | 750 | 21 | 26 | -3.111 | 0.002 | 0.004 |
|  | 1250 | 21 | 230 | 3.980 | 0.000 | 0.000 |
|  | 1750 | 21 | 193 | 2.694 | 0.007 | 0.009 |

**Table 3**. Wilcoxon signed rank tests comparing the change in tapping asynchrony between high and low roughness conditions at each time point for Experiments 1A and 1B.

|  | Time Point | N | W | z | p | p (corrected) |
| --- | --- | --- | --- | --- | --- | --- |
| Experiment 1A (online) | 250 | 67 | 1175.5 | 0.228 | 0.820 | 0.820 |
|  | 750 | 67 | 1400.6 | 1.634 | 0.102 | 0.409 |
|  | 1250 | 67 | 1197 | 0.362 | 0.717 | 0.820 |
|  | 1750 | 67 | 1001 | -0.862 | 0.389 | 0.777 |
| Experiment 1B (in-lab) | 250 | 21 | 94 | -0.747 | 0.455 | 0.768 |
|  | 750 | 21 | 150 | 1.199 | 0.230 | 0.768 |
|  | 1250 | 21 | 127 | 0.400 | 0.689 | 0.768 |
|  | 1750 | 21 | 107 | -0.295 | 0.768 | 0.768 |

**Table 4**. One-sample Wilcoxon tests comparing the change in tapping asynchrony in each condition and time point to zero for Experiments 2A and 2B.

|  | Time Point | N | W | z | p | p (corrected) |
| --- | --- | --- | --- | --- | --- | --- |
| Experiment 2A (online) | 250 | 49 | 398 | -2.134 | 0.033 | 0.044 |
|  | 750 | 49 | 333 | -2.780 | 0.005 | 0.011 |
|  | 1250 | 49 | 920 | 3.059 | 0.002 | 0.009 |
|  | 1750 | 49 | 733 | 1.199 | 0.231 | 0.231 |
| Experiment 2B (in-lab) | 250 | 20 | 7 | -3.659 | 0.000 | 0.001 |
|  | 750 | 20 | 58 | -1.755 | 0.079 | 0.106 |
|  | 1250 | 20 | 210 | 3.920 | 0.000 | 0.000 |
|  | 1750 | 20 | 131 | 0.971 | 0.332 | 0.332 |

**Table 5**. Wilcoxon signed rank tests comparing the change in tapping asynchrony between loud and soft distractors at each time point for Experiments 2A and 2B.

|  | Time Point | N | W | z | p | p (corrected) |
| --- | --- | --- | --- | --- | --- | --- |
| Experiment 2A (online) | 250 | 49 | 493 | -1.189 | 0.239 | 0.239 |
|  | 750 | 49 | 287 | -3.238 | 0.001 | 0.005 |
|  | 1250 | 49 | 860 | 2.462 | 0.014 | 0.028 |
|  | 1750 | 49 | 744 | 1.308 | 0.191 | 0.235 |
| Experiment 2B (in-lab) | 250 | 20 | 77 | -1.045 | 0.296 | 0.395 |
|  | 750 | 20 | 18 | -3.248 | 0.000 | 0.005 |
|  | 1250 | 20 | 174 | 2.576 | 0.010 | 0.020 |
|  | 1750 | 20 | 121 | 0.597 | 0.550 | 0.550 |

**Table 6**. One-sample Wilcoxon tests comparing the change in tapping asynchrony in each condition and time point to zero for Experiments 3A and 3B.

|  | Time Point | N | W | z | p | p (corrected) |
| --- | --- | --- | --- | --- | --- | --- |
| Experiment 3A (online) | 250 | 52 | 664 | -0.227 | 0.820 | 0.820 |
|  | 750 | 52 | 491 | -1.803 | 0.071 | 0.285 |
|  | 1250 | 52 | 637 | -0.474 | 0.626 | 0.820 |
|  | 1750 | 52 | 585 | -0.947 | 0.344 | 0.687 |
| Experiment 3B (in-lab) | 250 | 21 | 105 | -0.365 | 0.715 | 0.821 |
|  | 750 | 21 | 65 | -1.755 | 0.079 | 0.158 |
|  | 1250 | 21 | 170 | 1.894 | 0.058 | 0.158 |
|  | 1750 | 21 | 109 | -0.226 | 0.821 | 0.821 |

**Table 7**. Wilcoxon signed rank tests comparing the change in tapping asynchrony between large and small pitch shift at each time point for Experiments 3A and 3B.

|  | Time Point | N | W | z | p | p (corrected) |
| --- | --- | --- | --- | --- | --- | --- |
| Experiment 3A (online) | 250 | 52 | 730 | 0.373 | 0.709 | 0.870 |
|  | 750 | 52 | 328 | -3.288 | 0.001 | 0.004 |
|  | 1250 | 52 | 779 | 0.820 | 0.412 | 0.825 |
|  | 1750 | 52 | 671 | -0.164 | 0.870 | 0.870 |
| Experiment 3B (in-lab) | 250 | 21 | 134 | 0.643 | 0.520 | 0.694 |
|  | 750 | 21 | 32 | -2.902 | 0.004 | 0.015 |
|  | 1250 | 21 | 181 | 2.277 | 0.023 | 0.046 |
|  | 1750 | 21 | 126 | 0.365 | 0.715 | 0.715 |
